# Oral health status among orphan and non-orphan children in Mashhad: a case-control study

**DOI:** 10.25122/jml-2021-0127

**Published:** 2022-09

**Authors:** Razie Meshki, Leila Basir, Shiva Motaghi, Maryam Kazempour

**Affiliations:** 1 Department of Pedodontics, Dental School, Ahvaz Jundishapur University of Medical Sciences, Ahvaz, Iran

**Keywords:** orphanage, oral health, dental caries

## Abstract

Developing and modifying the policies of orphanages requires the availability of accurate information. This study aimed to compare caries and oral hygiene status among orphan and non-orphan children. This descriptive cross-sectional study took place in governmental orphanages and schools of both genders in Mashhad, Iran. The prevalence of caries was assessed using the Decayed, Missing and Filled Tooth (DMFT) index, and oral health status was assessed using the Oral Hygiene Index – Simplified (OHI-S). The results were statistically analyzed using a t-test and Chi-Square test. The mean DMFT was 3.36 in orphans and 2.10 in non-orphan children, which was not statistically significant (p=0.6). The mean dmft was 9.01 in the orphan group and 5.26 in the non-orphan group, which was statistically significant (p=0.003). The average OHI-S index was 2.30 in the group of orphan children and 1.05 in the non-orphan children, presenting a statistically significant difference (p=0.00). The prevalence of caries, especially in deciduous teeth, was high among orphan children. The oral health status of these children was worse than that of children living at home. Consequently, there is a need for proper planning to promote the oral health of children living in orphanages.

## INTRODUCTION

Oral health is a key component of general health, with the most common oral disease being dental caries [1]. Unfortunately, despite tooth decay being a global issue, no clear plan has been successfully implemented to eradicate the disease. In order to control caries in society, it is necessary to provide health services, social support for prevention plans, and an assessment system [2].

The World Health Organization (WHO) recommends conducting general oral health surveys. In fact, the first step in the process of designing health programs is to assess the needs of society. As a result, the problem is identified, and the extent and severity determined. The needs assessment also provides an overview of the community that will help determine the causes of the problem [3].

Today, many children lose their families due to issues such as financial poverty, cultural problems, mental health, or other factors and encounter difficult situations. An orphan is a child who has lost both parents or has been abandoned by them [4]. Orphan children are exposed to a variety of harms, both in terms of family deprivation and poor living conditions in boarding schools and care centers [5]. Children in orphanages have a high prevalence of caries, dental trauma, and gingivitis, which may be due to overcrowding, lack of adequate facilities, lack of hygiene, mental stress, and poor diet [6]. Orphan and neglected children also suffer from a number of emotional and psychological disorders that reduce their cooperation in health education [7]. This decrease refers both to reduced learning and reduced performance of health tasks [8].

Therefore, to improve health indices, target groups should be identified, and the causes of oral diseases should be eliminated. The results of several studies showed that orphan children were significantly worse off in medical and dental conditions [9, 10]. Children living in orphanages are at greater risk for dental and gingival problems due to psychological problems and lack of parental supervision [4]. Therefore, the society can focus on prevention and treatment options for this target group.

Additionally, this research provides basic information for health experts to reduce the rate of the indices by focusing on the causes, eliminating them, and bringing the outcomes closer to the world's standards. Considering that no study has been conducted on the oral health status of orphans welfare in Mashhad recently, this study was designed to investigate the oral health of orphans.

## MATERIAL AND METHODS

The present cross-sectional study evaluated children from orphanages belonging to government and children of school age living with their parents in Mashhad, Iran. The study was conducted from July 2020 to January 2021.

The sample size for the present study was obtained using the following equation [11].


$$N=\frac{Z(\alpha /2)2*p(1-p)}{E2}$$

Where, Z_α/2_ = normal deviate for two-tailed alternative hypothesis at a level of significance; p = prevalence or proportion of event of interest for the study; E = precision or margin of error.

The sample size was calculated from a previous study [12] with a confidence interval of 95%, a margin of error of 5%, and an anticipated non-response rate of 10%, resulting in a sample of at least 200. There were two registered orphanages in Mashhad. All the children from both centers who met the inclusion criteria were included in the study. Two hundred and twenty-two orphans aged 7–12 years who permanently lived in the orphanages were considered. An equal number of children from four schools in the nearby orphanage area were included in the control group. A simple randomization method and a table of random numbers were used. Every school child was assigned a number. If the selected number was even, the school child was allocated to the study group, and if it was odd, the school child was excluded from the study. Both orphan and non-orphan children were similar in gender distribution and mean age. The exclusion criteria were age less than 7 years and more than 12 years, lack of cooperation in the examination, systemic disorders, mental or other behavioral disorders, enamel and dentin defects other than tooth decay, and the use of orthodontic devices. Intraoral examinations were performed by a trained examiner (postgraduate pediatric dentistry student) using a mouth mirror and personal protective barriers (gloves and masks). The data were recorded on a standard WHO form. The status of teeth caries was recorded by World Health Organization (WHO) criteria including the indices of Decayed, Missing, Filled Teeth/Surfaces (DMFT/DMFS) for the permanent teeth and the decayed, missing and filled teeth/surfaces (dmft/dmfs) for the primary teeth [13]. The oral hygiene status was recorded using the oral hygiene index (OHI-S), described by Vermillion and Greene [14]. The values of debris and calculus were determined by examining the facial surface of teeth 11, 16, 26, and 31 and the lingual surface of teeth 36 and 46. For each person, the OHI-S score was calculated by dividing the sum of debri and calculus values by the number of surfaces. Based on the calculated OHI-S values, oral health status was grouped into two categories: favorable (score 0 to 3) and unfavorable (3 to 6), to simplify the comparison. The reliability of the examiner was evaluated by test re-test with the re-examination of 11% of the samples. T-test were used to analyze DMFT/DMFS, dmft/dmfs and OHI-S scores. A p-value<0.05 was considered to be significant.

## RESULTS

This study was performed on 444 children aged 7–12 years. The first group consisted of 222 orphan children from orphanages with an average age of 8.87 years. The second group included 222 non-orphan children, with a mean age of 9.08. The difference between their mean ages was not statistically significant. According to t-test analysis, the mean DMFT was 3.36 in orphan children and 2.10 in non-orphan children, which was not statistically significant (p=0.6). The mean dmft was 9.01 in the orphan group and 5.26 in the non-orphan group, which was statistically significant (p=0.003). The next index examined was the OHI-S index. The average OHI-S index was 2.30 in the group of orphan children and 1.05 in the other group, presenting a statistically significant difference (p=0) (Table 1).

**Table 1:** Independent Samples Test.

	Group	N	Mean	Std. Deviation	p-value
Age	Orphan	222	8.87	1.648	0.754
	Non-orphan	222	9.08	1.636	
DMFT	Orphans	222	3.36	1.861	0.607
	Non-orphan	222	2.10	1.843	
dmft	Orphans	222	9.01	3.858	0.003
	Non-orphan	222	5.26	3.413	
OHI-S	Orphans	222	2.30	.689	0.000
	Non-orphan	222	1.05	.990	

According to Table 2, only 11% of orphan children had favorable oral health (OHIS=0-1), while 88% had unfavorable oral health (OHIS=1-3). Of the non-orphan group, 66% had favorable oral health status, and 33% had unfavorable oral health status.

**Table 2:** Oral hygiene status.

Group	OHI-S	N (%)
**Orphan**	Favorable	**26 (11%)**
	Unfavorable	**196 (88%)**
**Non-orphan**	Favorable	**148 (66%)**
	Unfavorable	**74 (33%)**

In this study, 61.7% of orphan children were girls, and 38.3% were boys. In the non-orphan group, 58.1% of the participants were girls, and 41.9% were boys. According to the Chi-Square test, the difference between the number of girls and boys in the two groups was not significant. The mean DMFT and dmft in both groups were significantly higher in boys than girls (Tables 3 and 4). Although the OHI-S index in orphan boys was higher than in orphan girls, this difference was not statistically significant. The OHI-S index was higher in non-orphan girls than non-orphan boys, but this difference was not statistically significant (Table 5).

**Table 3:** Comparison of DMFT index between girls and boys.

Group	Gender	Mean	Std. Deviation	p-value
Orphan	F	3.00	1.627	137
	M	3.95	2.064	85
	Total	3.36	1.861	222
Non-orphan	F	2.10	1.785	129
	M	2.11	1.931	93
	Total	2.10	1.843	222
Total	F	2.56	1.761	266
	M	2.99	2.194	178
	Total	2.73	1.955	444

**Table 4:** Comparison of dmft index between girls and boys.

Group	Gender	Mean	Std. Deviation	p-value
Orphan	F	8.60	3.849	137
	M	9.68	3.799	85
	Total	9.01	3.858	222
Non-orphan	F	4.69	2.936	129
	M	6.04	3.861	93
	Total	5.26	3.413	222
Total	F	6.70	3.949	266
	M	7.78	4.234	178
	Total	7.14	4.095	444

**Table 5:** Comparison of OHI-S index between girls and boys.

Group	Gender	Mean	Std. Deviation	p-value
orphan	F	2.30	.690	137
	M	2.31	.690	85
	Total	2.30	.689	222
Non-orphan	F	1.02	.935	129
	M	1.10	1.064	93
	Total	1.05	.990	222
Total	F	1.68	1.039	266
	M	1.67	1.087	178
	Total	1.68	1.057	444

## DISCUSSION

In this study, we evaluated the oral health and prevalence of dental caries in children from the Mashhad orphanages compared to non-orphan children. Orphaned children had regular check-ups and more detailed health records. This may be due to the small clinics in each orphanage where children regularly visited specific physicians and dentists. In previous studies, the mean rate of dmft was 4.72 in 6–7 years old children in Mashhad [15]. In contrast, in our study, this rate was 5.26 for non-orphan children and 9.1 for orphan children. Furthermore, the almost double increase of dmft in orphan children indicates the need for more education and attention to better oral hygiene and care, especially in the period of primary dentition. The lower mean in DMFT of orphan children (3.36) was higher than the mean of non-orphan children (2.1), with a lack of significant differences between them (p=0.6). This indicates better health at older ages in orphanages and emphasizes that younger children should be given more attention. The interpretation is that children living in orphanages have a predetermined diet and consume fewer snacks such as sweets and cakes than children living in a family. Children in orphanages did not have good oral hygiene and were responsible for brushing themselves; therefore, the effect of diet on the reduction of DMFT may have been far greater than the effect of oral hygiene.

A previous study by Mazhari et al. (2006) in Mashhad orphanages reported the mean of dmft 3.4 and the mean of DMFT 1.37, which presented lower values than those reported in the present study. Contrary to their findings, our study revealed that the mean of dmft and DMFT in boys was significantly higher than in girls. Our research revealed no significant difference in oral health index between boys and girls. This issue was not studied in the study of Mazhari et al. [12].

The prevalence of caries in both groups was higher than in developed countries, consistent with previous studies in Iran [12, 15–17]. According to the World Health Organization, the mean of DMFT was 1.2–2.6 in developed countries [18]. Education on the prevention of dental caries should be increased for all children in the community, especially in vulnerable groups. These results were confirmed by other studies in Mexico City and Riyadh, stating that the presence of caries is high in orphan children, as they only go to the dentist when in need of treatment for toothaches [9, 19]. However, it is demonstrated that lower IQ levels, parent deprivation, and institutionalization are risk factors for the development of caries in orphan children [20].

The mean of OHI-S was significantly higher in orphaned children than in non-orphan children. Only 11% of the orphan children had favorable oral health, in contrast to 66% of the non-orphan children. This result was predictable by children having more access to oral hygiene items at home and having increased parental supervision over their oral hygiene. In agreement with our results, previous studies reported that the rate of oral hygiene among orphans is lower than among children living with their parents [9, 19, 21]. Al-Jobair et al. considered improper brushing technique as one of the critical factors of poor oral hygiene [9]. Camacho et al. examined the amount of plaque on the surface of the teeth of children living in the orphanages, revealing that the oral health of these children is much lower than that of children living at home [19].

## CONCLUSION

Due to the higher prevalence and severity of the decay of deciduous teeth in children living in orphanages and their unfavorable oral health, there is a clear need for planning and policy-making to promote the oral health of these children.

## Personal thanks

The authors would like to thank the Ministry of Health and State Welfare Organization of Iran for their cooperation. Appreciation is also extended to the orphanages' administration, school authorities, students, and parents.
